# Surface Passivation of III–V GaAs Nanopillars by Low-Frequency Plasma Deposition of Silicon Nitride for Active Nanophotonic Devices

**DOI:** 10.1021/acsaelm.2c00195

**Published:** 2022-07-01

**Authors:** Bejoys Jacob, Filipe Camarneiro, Jérôme Borme, Oleksandr Bondarchuk, Jana B. Nieder, Bruno Romeira

**Affiliations:** † INL − International Iberian Nanotechnology Laboratory, Ultrafast Bio- and Nanophotonics group, Av. Mestre José Veiga s/n, 4715-330 Braga, Portugal; ‡ INL − International Iberian Nanotechnology Laboratory, 2D Materials and Devices group, Av. Mestre José Veiga s/n, 4715-330 Braga, Portugal; § INL − International Iberian Nanotechnology Laboratory, Advanced Electron Microscopy, Imaging and Spectroscopy Facility, Av. Mestre José Veiga s/n, 4715-330 Braga, Portugal

**Keywords:** nanopillars, III−V semiconductors, GaAs/AlGaAs, silicon nitride, surface passivation, low-frequency plasma deposition, internal quantum efficiency, nanoLEDs

## Abstract

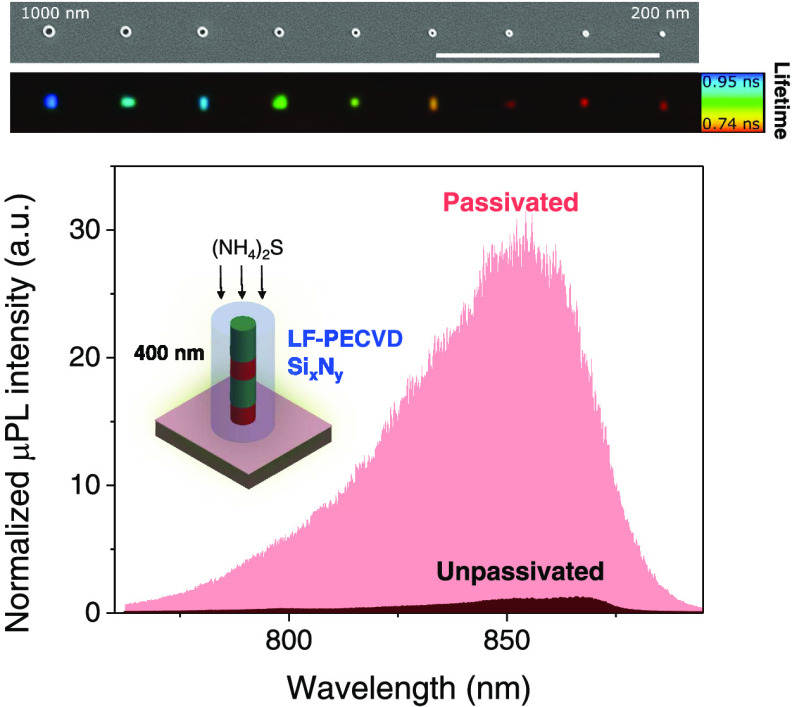

Numerous efforts have been devoted to improve the electronic and optical properties of III–V compound materials via reduction of their nonradiative states, aiming at highly efficient III–V sub-micrometer active devices and circuits. Despite many advances, the poor reproducibility and short-term passivation effect of chemical treatments, such as sulfidation and nitridation, requires the use of protective encapsulation methods, not only to protect the surface, but also to provide electrical isolation for device manufacturing. There is still a controversial debate on which combination of chemical treatment and capping dielectric layer can best reproducibly protect the crystal surface of III–V materials while being compatible with readily available semiconductor-foundry plasma deposition methods. This work reports on a systematic experimental study on the role of sulfide ammonium chemical treatment followed by dielectric coating (either silicon oxide or nitride) in the passivation effect of GaAs/AlGaAs nanopillars. Our results conclusively show that, under ambient conditions, the best surface passivation is achieved using ammonium sulfide followed by encapsulation with a thin layer of silicon nitride by low-frequency plasma-enhanced chemical deposition. Here, the sulfurized GaAs surfaces, high level of hydrogen ions, and low-frequency (380 kHz) excitation plasma that enable intense bombardment of hydrogen, all seem to provide a combined active role in the passivation mechanism of the pillars by reducing the surface states. As a result, we observe up to a 29-fold increase of the photoluminescence (PL) integrated intensity for the best samples as compared to untreated nanopillars. X-ray photoelectron spectroscopy analysis confirms the best treatments show remarkable removal of gallium and arsenic native oxides. Time-resolved micro-PL measurements display nanosecond lifetimes resulting in a record-low surface recombination velocity of ∼1.1 × 10^4^ cm s^–1^ for dry-etched GaAs nanopillars. We achieve robust, stable, and long-term passivated nanopillar surfaces, which creates expectations for remarkable high internal quantum efficiency (IQE > 0.5) in nanoscale light-emitting diodes. The enhanced performance paves the way to many other nanostructures and devices such as miniature resonators, lasers, photodetectors, and solar cells, opening remarkable prospects for GaAs active nanophotonic devices.

## Introduction

A wide range of nanoscale light sources have been reported employing III–V materials as the gain medium.^1,2^ These semiconductor compound materials are crucial for the fabrication of miniaturized optical sources, such as nanoscale light-emitting diodes (nanoLEDs)^3^ and nanolasers,^4^ of growing importance for compact photonic integrated circuits (PICs) needed in optical data communications,^5^ optical computing including neuromorphic computing,^6−8^ and sensing and spectroscopy,^1,2,9^ as well as medical diagnosis applications. Noteworthy, in the past few years, remarkable developments in nanoLEDs have been made using either semiconductor III–V or III–V on silicon materials. The approach for miniaturization relies on the use of cavities such as photonic crystals,^10^ metal-dielectric,^11^ or plasmonic,^3^ thus enabling the realization of wavelength- and sub-wavelength-scale devices. Alternative nanoLED architectures also include the use of other material systems, such as fin-shaped semiconductors,^12^ even though extreme current densities are required in this case. These advances are creating expectations that nanoLEDs can be both efficient and fast, thereby capable of outperforming nanolasers.^13,14^ However, to this date, the external quantum efficiency (EQE) at room temperature of III–V nanoLEDs remains limited to values below 1%, resulting in ultralow output powers (in the nanowatt or even picowatt range),^3,10,11^ which makes nanoLEDs challenging for practical optical systems. Taking the example of III–V nanopillars and neglecting losses related with metallic structures in metal-dielectric or plasmonic nanocavities, the main reasons for the extremely low EQEs are two-fold. First, coupling the light output efficiently to a nano-waveguide,^11^ or a plasmonic waveguide,^3^ remains a challenge when the area of the light source is reduced to deep sub-micrometer.^2^ Second, at these small scales, nonradiative effects in III–V materials, specifically surface-related properties, become more important as the surface-to-volume ratio increases substantially. In this work, we devote our attention to the role of the nonradiative effects in the performance of III–V gallium arsenide (GaAs) light-emitting sub-wavelength devices.

Among the wide range of III–V materials available for active nanophotonic devices, GaAs/AlGaAs is one of the most studied and a key compound material for photonics,^4,15−18^ providing optical emission and absorption in a wide range of wavelengths spanning from the visible to near-infrared (NIR). GaAs has recently been notable in many photonic applications such as 3D sensing using GaAs-based lasers, NIR-LEDs, and visible red–orange–yellow LEDs for displays. However, the surfaces of GaAs-based materials and their interfaces with dielectrics tend to host large densities of electronically active defects (dangling bonds).^19^ As a result, at ambient conditions, an oxide layer is formed on the surface of GaAs (e.g., Ga_2_O_3_ and As_2_O_3_), which leads to charge trapping.^20^ Importantly, when semiconductors are nanostructured, namely, using top-down dry etching, the plasma reactive etching process can induce additional surface damages,^21^ such as surface roughness due to ion bombardment, surface contamination due to polymer deposition, or surface stoichiometry change due to preferential etching. Overall, this results in charge trapping effects (i.e., nonradiative active centers), leading in the case of GaAs nanoscale LEDs to extremely short lifetimes (sub-100 ps)^16^ and ultralow efficiencies.^11,22^

A wide range of methods have been reported for passivating GaAs surfaces of micro- and nanoscale structures and devices.^10,17,18^ One technologically challenging and expensive method is the epitaxial growth of a high band gap layer on the GaAs surface.^23−28^ The high band gap layer reduces the surface trap density since it prevents carriers in GaAs from accessing the surface states and thus reduces the photoluminescence (PL) decay rate. On a second approach, chemical passivation, including nitridation^29,30^ and sulfidation^20,31−34^ by wet chemistry, is an inexpensive and widely used method. Sulfidation, for example, has proven to be effective in removing the native oxides and elemental arsenic from the surface by creating an S termination on the semiconductor surface.^20,31,32^ Still, this termination tends to be unstable when exposed to air or water and the passivation procedure is strongly dependent on the chemical composition, light, and temperature conditions, which makes it difficult to achieve reproducible results. Nitridation in bulk GaAs samples has been recently shown to be more robust and resistive to air over about 100 h.^30^ In GaAs optical wavelength-sized optical structures (disk resonators),^35^ wet nitridation revealed to substantially increase their optical quality factor. But in all scenarios, protective layers are still needed not only to prevent the sulfide or nitride layer’s degradation (due to oxidation or other environmental effects) but also to provide electrical isolation for optoelectronic device manufacturing.

Several deposition methods can produce dielectric films such as silicon oxide (SiO_2_), silicon nitride (Si_3_N_4_), and alumina (Al_2_O_3_), with excellent properties, including atomic layer deposition (ALD) and plasma-enhanced chemical vapor deposition (PECVD). Interestingly, a number of studies suggest that the surface passivation can be highly sensitive to the structure and composition of the semiconductor-dielectric interface, and the interface formation process may depend on the hydrogen content, stoichiometry, and density of the ALD- and PECVD-fabricated films and also on subsequent temperature treatments.^36^ As a result, the protective layers can not only prevent degradation of the surface but play an active role on the passivation effect.^37^ Recently, it has been reported that not only the type of protective film but also the frequency of the plasma deposition (specifically lower RF excitation) can play an important role in the passivation of *n*-type GaAs electronic devices,^38^ due to the ionic bombardment inherent to the low-frequency plasma. Nevertheless, its impact in the optical properties of GaAs-based semiconductors and nanostructures has been overlooked and, to our knowledge, totally unexplored.

In this study, we report on an experimental investigation to identify which combination of chemical passivation and dielectric protective film layer could best reproducibly passivate and protect the crystal surface of III–V materials, while being compatible with readily available semiconductor-foundry plasma deposition methods. Specifically, we present a systematic experimental study that investigates the role of the sulfide ammonium chemical treatment followed by various dielectric coatings (SiO*_x_* or Si*_x_*N*_y_*) by either low-frequency or high-frequency PECVD in the surface passivation effect of unintentionally doped GaAs/AlGaAs compound semiconductor nanopillars. Our results conclusively show that, under ambient conditions, the best passivated surfaces of sub-micrometer deeply etched GaAs/AlGaAs nanopillars are achieved using a combination of ammonium sulfide followed by encapsulation with a thin-film layer of Si_x_N_y_ (∼80 nm) deposited by low-frequency PECVD at 300 °C. For this surface treatment, a remarkable 29-fold enhancement of the PL intensity is achieved for the best samples as compared to untreated nanopillars. We observe a robust, stable, and long-term (>10 months) passivation effect for nanopillars ranging from 200 nm to 1 μm. The quality of the passivation treatment can be quantified by the minimum amount of surface defects formed by the native oxides on the GaAs surface and this has been analyzed by X-ray photoelectron spectroscopy. The measurements show successful removal of gallium and arsenic native oxides for the best treatment using sulfurization of GaAs pillars immediately followed by HF-PECVD Si*_x_*N*_y_* deposition, which is in line with the PL measurements. Time-resolved PL measurements reveal that the lifetimes of the best passivated nanopillars can reach a lifetime of ∼1 ns, leading to estimations of a record-low surface recombination velocity of ∼1.1 × 10^4^ cm s^–1^ for dry-etched GaAs-based nanopillars. This value compares some of the best passivated core–shell GaAs/AlGaAs nanowires (1.7 × 10^3^ to 1.1 × 10^4^ cm s^–1^) of similar width dimensions. However, our method uses a conventional semiconductor-foundry industrial-ready PECVD deposition method instead of challenging and expensive epitaxial growth methods.^27^ These results demonstrate the impact of surface passivation on the internal quantum efficiency (IQE) of passivated GaAs-based light-emitting pillars that could reach an IQE > 0.5. Our results pave the way for III–V GaAs active nanophotonic devices such as nanoLEDs and nanolasers operating at room temperature with large efficiencies and other relevant sub-micrometer structures such as nano-waveguides and miniature resonators.

## Experimental Section

### Fabrication of GaAs/AlGaAs Nanopillars

**Figure 1:**
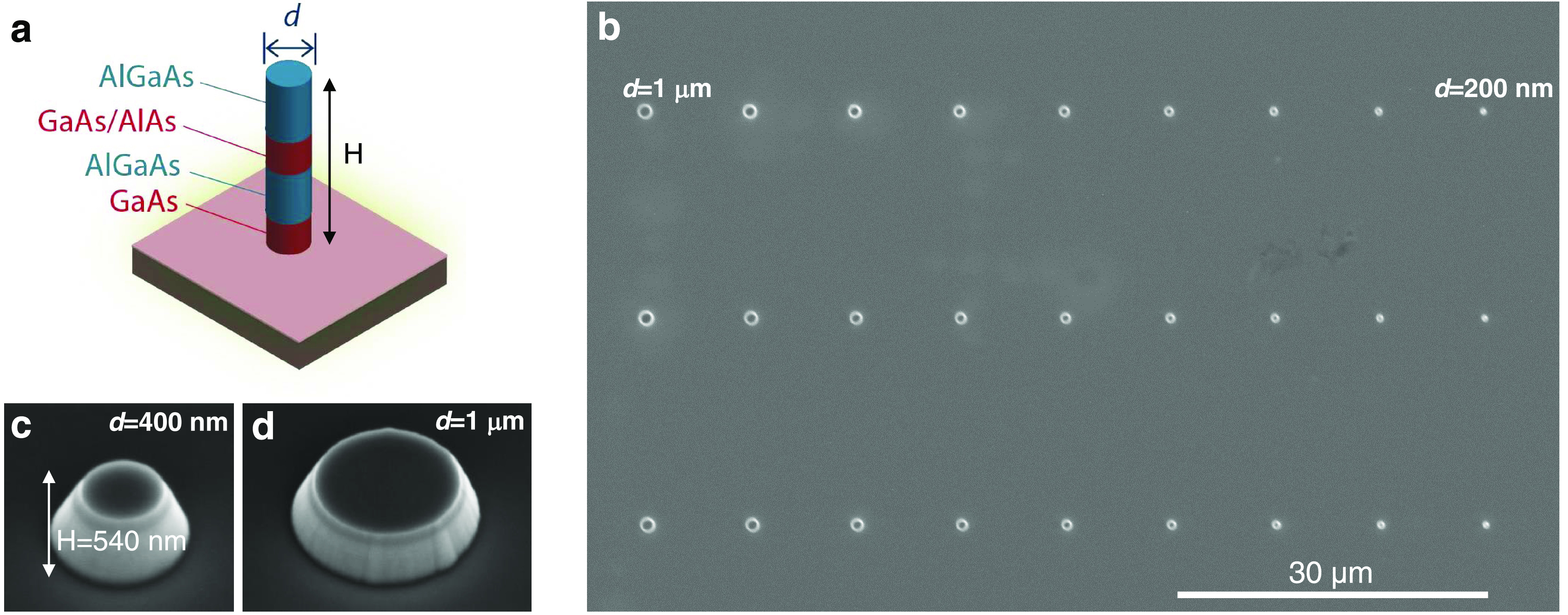
Scheme and fabricated III–V semiconductor GaAs/AlGaAs nanopillars. (a) Schematic of a GaAs/AlGaAs nanopillar. SEM images of (b) GaAs/AlGaAs pillar array, (c) 400 nm wide nanopillar, and (d) 1 μm wide micropillar.

A systematic experimental study was performed to investigate the passivation effect on AlGaAs/GaAs/AlGaAs nanopillars. The semiconductor layer stack (Figure 1a) was composed from top to bottom, by 150 nm of AlGaAs (30% Al), 52 nm of a GaAs-based compound material consisting of a GaAs (20 nm)/AlAs (3 nm)/GaAs (6 nm)/AlAs (3 nm)/GaAs (20 nm) double barrier quantum well (DBQW) nanostructure, 150 nm of AlGaAs (30% Al), and 300 nm of GaAs, all not intentionally doped, and grown by molecular beam epitaxy on a GaAs substrate. The selection of the GaAs-DBQW nanostructure is motivated by its quantum resonant tunneling phenomenon for applications in electrically pumped nonlinear LED sources of relevance for neuromorphic nanophotonic computing.^8^ In this work, we are mainly interested in the passivation effect on the GaAs/AlGaAs layer stack to achieve efficient light emission. The fabrication of the nanopillars involved nanopatterning via electron beam lithography using a Vistec 5200 ES 100 kV tool. The pillars were dry-etched until *H* ∼ 0.54 μm depth (*H* is the height of the nanopillar) using inductively coupled plasma (ICP) in an SPTS ICP machine (the nanofabrication description can be found in Supporting Information S1).

Figure 1b displays the scanning electron microscope (SEM) image of representative fabricated semiconductor pillars. The samples contained pillars with dimensions ranging from 200 nm to 1 μm width, *d*, organized in arrays spaced by at least 10 μm so that the emission could be collected and analyzed individually from each single pillar. On the same sample (not shown), micropillars with dimensions ranging from 3 to 8 μm width were also fabricated. Figure 1c shows the SEM picture of a *d* ∼ 400 nm wide circular nanopillar, and Figure 1d shows an example of a micropillar (1 μm width). As a result of the dry-etching step, the nanopillars typically displayed sloped sidewall features with an angle of ∼17°.

### Surface Passivation Treatments

A set of identical samples containing micro- and nanopillar arrays were fabricated as discussed previously. After fabrication of the nanopillars, the surface passivation entailed the following six main treatment procedures (Table 1). Treatment #1 was a sulfur treatment only consisting of a 20% ammonium sulfide solution that was further diluted [H_2_O/(NH_4_)_2_S (10:1)], where samples were dipped for 5 min at 65 °C under dark conditions. In treatment #2, the samples were submerged in ammonium sulfide solution, similarly as described in treatment #1. Then, immediately after the sulfur, a thin capping layer of SiO*_x_* was deposited by high-frequency (RF excitation source of 13.56 MHz) PECVD. In treatment #3, the thin capping layer of SiO*_x_* was deposited immediately after the sulfur using low-frequency (RF excitation source of 380 kHz) PECVD. Here, the RF plasma was tuned well below the ion transit frequency (estimated ∼2 MHz). In treatment #4, immediately after sulfurization, a thin capping layer of Si*_x_*N*_y_* was deposited by high-frequency (13.56 MHz) PECVD. In treatment #5, instead of high-frequency PECVD, the Si*_x_*N*_y_* deposition was performed by low-frequency (RF excitation source 380 kHz) PECVD. Lastly, in treatment #6, the samples were coated with a thin capping layer of Si*_x_*N*_y_* deposited also by low-frequency (380 kHz) PECVD but without employing the ammonium sulfide solution pretreatment. All film depositions were performed with a substrate temperature of 300 °C. A complete description of treatments #1–#6 can be found in Supporting Information S2. For the purpose of comparing both passivated and unpassivated pillars under the same fabrication processing conditions, for each passivation treatment (Table 1), an unpassivated sample of pillars was simultaneously fabricated and left uncoated without any sulfurization treatment.

**Table 1:**
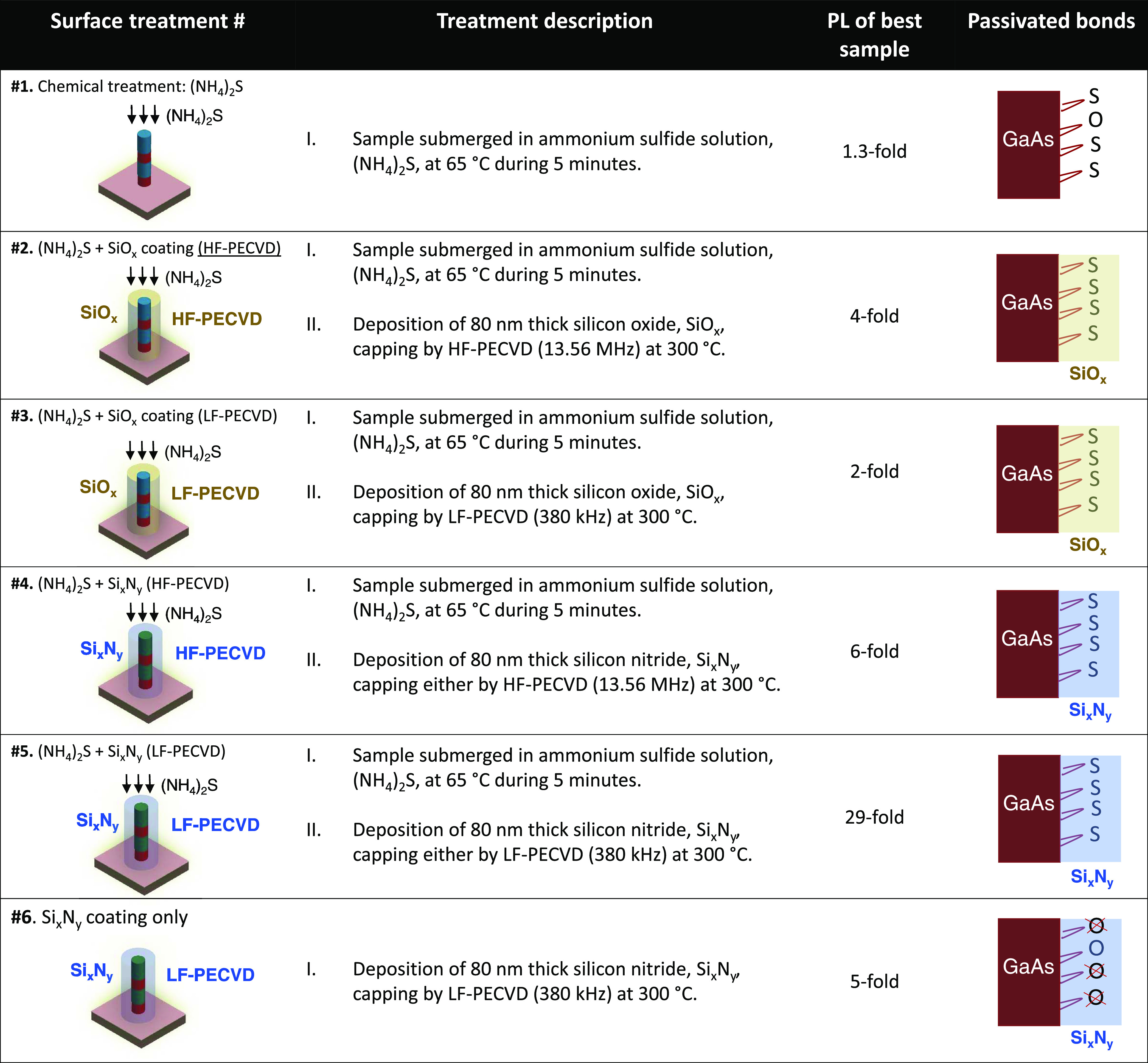
Summary of the Main Surface Treatments Showing the Treatment Description, Value of PL Improvement as Compared with Unpassivated Sample, and Schematic of the Passivated Bonds at the Surface of GaAs Materials for Various Treatments^a^

a The value of the PL integrated intensity improvement was taken for the best samples for the case of a 400-nm-wide nanopillar.

### Steady-State and Time-Resolved Micro-Photoluminescence

The photoluminescence of fabricated nanopillars and the effect of the respective surface passivation treatment (Table 1) were measured using a micro-photoluminescence (μPL) setup consisting of a Witec α 300R confocal microscopy system fiber-coupled to a UHTS300 spectrometer coupled to an Andor Peltier cooled CCD detector. In our measurements, we have used a continuous-wave laser at 532 nm wavelength (2.33 eV energy) under low pumping conditions. The optical emission from the pillars was collected using a 100× air objective with a high numerical aperture (Supporting Information S3). The PL decay was measured in a time-correlated single-photon counting (TCSPC) experimental setup described in Supporting Information S4. In short, the output of a pulsed laser diode at 561 nm (2.21 eV), with a pulse with a full width at half-maximum (FWHM) of ∼80 ps and a repetition rate of 50 MHz, was used for excitation of the micro- and nanopillars. The pillars were optically pumped using a 100× high numerical aperture oil immersion objective.

### Energy-Dispersive X-ray Spectroscopy (EDS) and X-ray Photoelectron Spectroscopy (XPS)

The quality of the passivation treatments can be quantified by the amount of surface defects formed by gallium and arsenic native oxides, Ga-O (Ga_2_O_3_) and As-O (As^3+^ and As^5+^), respectively. The analysis of these native oxides on the GaAs surface was performed using EDS and XPS. Initial surface characterization studies of fabricated samples employed EDS analysis with a scanning electron microscope (FEI NovaNanoSEM 650) equipped with an EDS system (detailed description in Supporting Information S6). XPS spectra were collected using an ESCALAB 250Xi system in UHV (<10^–9^ Torr). A monochromatic Al-Kα source (1486.6 eV) was used to analyze an area of 650 μm × 650 μm in the prepared samples. Since XPS spectra can be effectively collected using only thicknesses within 10 nm from the surface, for the XPS measurements, samples were prepared with deposited dielectric coatings with a thicknesses of ∼4 nm instead of ∼80 nm (a detailed description can be found in Supporting Information S7).

## Results and Discussion

### Steady-State PL Spectroscopy

The PL of the pillars was characterized in dependence of the applied chemical pretreatments, the dielectric coatings, and in dependence of the plasma frequency of the coating deposition, as summarized in Table 1. The table shows the value of PL improvements as compared with unpassivated samples taken for the best samples for the case of a 400 nm wide nanopillar. In summary, the chemical treatment with sulfurization showed only minor PL improvements (∼1.3-fold). The passivation with sulfurization followed by SiO*_x_* coatings (deposited either by LF- or HF-PECVD) showed an intermediate performance with PL improvements ranging from 2 to 4-fold. Lastly, passivation treatments using Si*_x_*N*_y_* coatings (either by LF- or HF-PECVD) displayed the best performance with PL improvements for the best samples ranging from 5 to 29-fold. Particularly, the results indicate that the best improvements (up to 29-fold) are achieved for nanopillars encapsulated with a layer of Si*_x_*N*_y_* deposited by low-frequency PECVD. Next, the PL results for each treatment #1–#6 are analyzed and discussed in detail.

**Figure 2:**
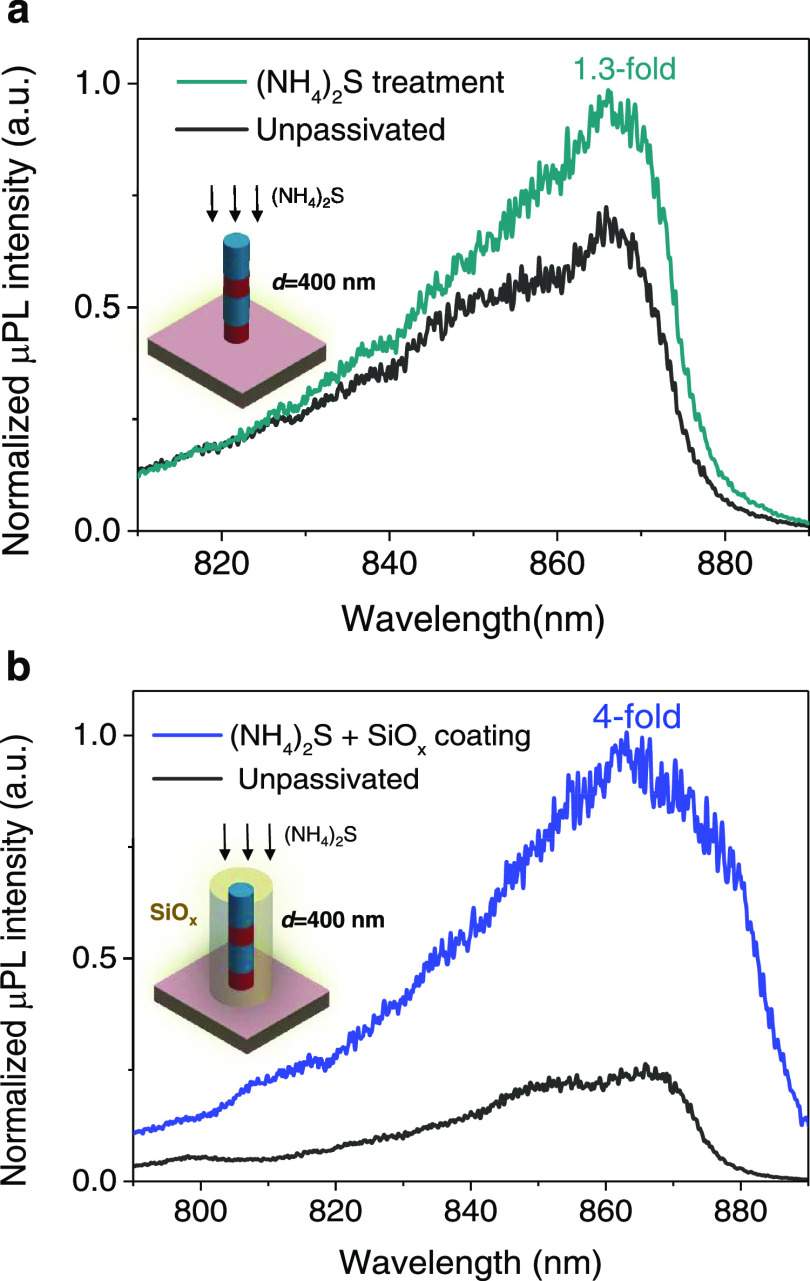
Continuous-wave photoluminescence measurement results at room temperature displaying a typical μ-PL spectrum from a single nanopillar with around 400 nm width for (a) unpassivated and sulfur passivation treatment passivation steps and (b) unpassivated and ammonium sulfide followed by SiO*_x_* coating deposited by HF-PECVD passivation treatment steps.

Figure 2a displays the typical μPL spectra of a representative 400-nm-wide nanopillar for treatment #1 (sulfurization only) showing the typical luminescence for both passivated and unpassivated cases. For this sulfurization treatment, neither sulfurization realized at room temperature (results not shown) nor at 65 °C (Figure 2a) revealed meaningful improvements. This can be expected since it is known that the reproducibility of sulfurization passivation treatments is strongly dependent on the temperature, light conditions, pH, and composition of the solution making it difficult to achieve reproducible results. Figure 2b shows the μPL spectrum for treatment #2 (sulfurization followed by SiO*_x_* coating). Interestingly, here, a 4-fold increase in the PL integrated intensity is observed. We note, however, that this result is much lower than the improvements shown for other III–V materials (e.g., InGaAs) using a similar procedure.^37^ An identical passivation treatment but using SiO*_x_* deposition by LF-PECVD instead (treatment #3, PL not shown) did not reveal substantial PL improvements (∼2-fold) as compared with unpassivated samples. This follows similar studies that consistently report that the passivation of GaAs shows the best results when Si*_x_*N*_y_* coating materials are employed, as previously shown in field-effect transistors,^39^ or terahertz emitter devices.^40^ This is attributed not only to the excellent source of hydrogen for further passivating the residual interface defect states that can be obtained when Si*_x_*N*_y_* is deposited by PECVD^39^ but also to the fact that Si*_x_*N*_y_* films coated in an initial clean surface can additionally participate directly in the formation of interfacial bonding at the GaAs surface in such a way as to reduce the density of defect sites.^41^

**Figure 3:**
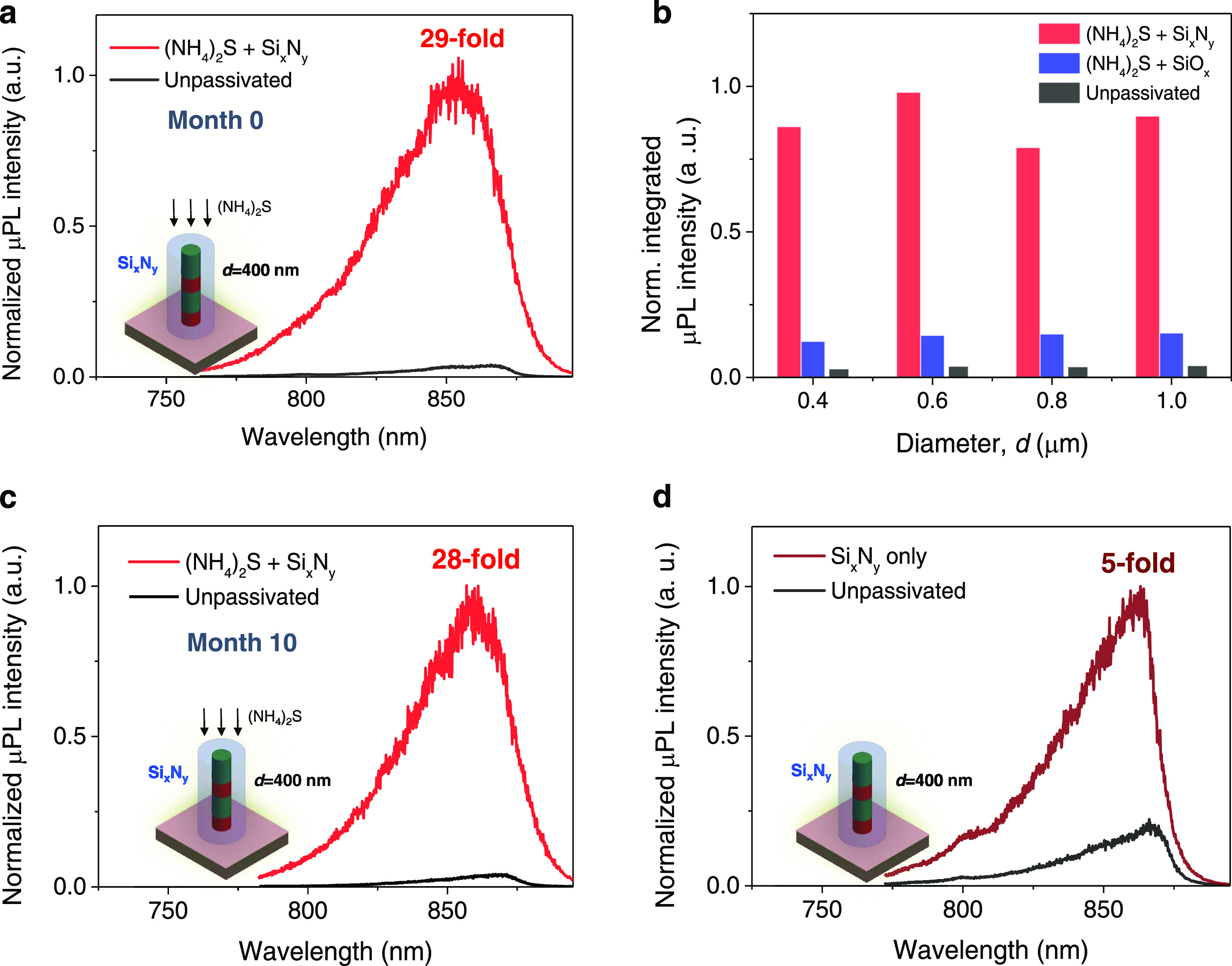
Experimental continuous-wave photoluminescence measurement results at room temperature. (a) Photoluminescence results displaying a typical μ-PL spectra from a single nanopillar with around 400 nm width for an unpassivated sample (black curve) and a sample treated with ammonium sulfide followed by Si*_x_*N*_y_* coating deposited by LF-PECVD (red curve). Inset shows a schematic of the best treatment displaying a pillar coated with Si*_x_*N*_y_* dielectric. (b) Normalized intensity as a function of the nanopillar width for all three untreated and passivation treatment cases for pillar diameters ranging from 400 nm to 1 μm. (c) Repeated PL measurements for the same samples shown in panel (a) after 10 months. (d) Photoluminescence results displaying the μ-PL spectra for unpassivated samples and samples using Si*_x_*N*_y_* coating by low-frequency PECVD, without chemical pretreatment.

Indeed, our tests reveal that all of the treatments using Si*_x_*N*_y_* layers show the best results as compared to SiO*_x_*. For example, treatment #4 (Si*_x_*N*_y_* by high-frequency PECVD) shows a PL improvement of up to 6-fold as compared to SiO*_x_*. Remarkably, when the Si*_x_*N*_y_* film is deposited by LF-PECVD immediately after sulfurization (treatment #5), up to a 29-fold PL intensity increase (measured in an identical 400 nm wide nanopillar) was achieved (red trace of Figure 3a) as compared to the unpassivated sample (black trace). Figure 3b shows a histogram of the integrated spectra summarizing the results from treatments #2 (blue), #5 (red), and untreated (black) pillars as a function of the pillar width. PL improvements were achieved for pillars ranging from 1 μm down to 400 nm, showing a PL enhancement ranging from 22 to 29-fold, respectively, as compared with untreated pillars. PL enhancements are observed also in the bulk surface region of the etched GaAs material and for micropillar-sized pillars (>1 μm) indicating an impressive passivation effect in either sub-micrometer/micrometer etched structures or bulk materials (S5, Figure S3). Noteworthy, Figure 3c presents measurements of the nanopillar sample shown in Figure 3a but recorded after 10 months (the samples were stored with regulated conditions at a temperature of 20 °C and a humidity of 40%). A similar PL (red trace) enhancement of ∼28-fold is achieved. The results indicate a stable and long-term passivation effect. The PL is compared with the same untreated sample shown in Figure 3a that was protected in month 0 with SiO*_x_* to avoid further oxidation (we note that this procedure did not affect the initial PL measured in Figure 3a, black trace).

Finally, Figure 3d displays the μPL spectra of an identical 400 nm pillar for the unpassivated case and for treatment #6, that is, using Si*_x_*N*_y_* coating by low-frequency PECVD only and without employing any chemical pretreatment. Improvements of the PL (∼5-fold) were achieved indicating that the low-frequency plasma indeed plays a role in the passivation effect. As a result, this dry-only single-step passivation method using low-frequency plasma shows a unique potential to be used in industrial environments for highly reproducible, simple, and cost-efficient passivation methods. Lastly, we note that when comparing the spectra from unpassivated and passivated samples, the emission wavelength peak for the passivated samples typically ranges from ∼854 to 858 nm. This emission is attributed to the central 52 nm DBQW GaAs active region and is consistent with the expected electron to heavy/light-hole band gap transitions from the 20 nm GaAs QW layers surrounding the AlAs barriers. The unpassivated or poorly passivated samples show emission mainly peaking at ∼865 nm corresponding to emission from the bottom GaAs region ∼190 nm, and therefore, close to the band-edge emission expected for a GaAs bulk material (∼872 nm; Supporting Information S5, Figure S3). This suggests that after successful passivation, a pronounced emission enhancement effect is achieved particularly for the GaAs-DBQW active material.

### EDS and XPS Analysis

The quality of passivation treatments can be further quantified by the amount of surface defects formed by the native oxides on the GaAs surface [here, Ga-O (Ga_2_O_3_) and As-O (As^3+^ and As^5+^)]. In this section, we focus our analysis on the removal of these native oxides by the treatments employing Si*_x_*N*_y_* layers that showed the best PL improvements. For initial surface characterization studies of PECVD Si*_x_*N*_y_* treatments, we used EDS in a scanning electron microscope system (see Supporting Information S6). For the pillars measured (pillar width 200 nm–1 μm), traces of oxygen were not identified (Figure S4), indicating a good passivation of PECVD Si*_x_*N*_y_* treatments. We note that, in the EDS analysis of our SEM system, it is challenging to quantify the presence of native oxides below 1 atomic percentage (atom %), in particular, light atoms. As a result, to quantify and compare the removal of gallium and arsenic oxides in various treatments, we focused our attention on samples measured by XPS.

**Figure 4:**
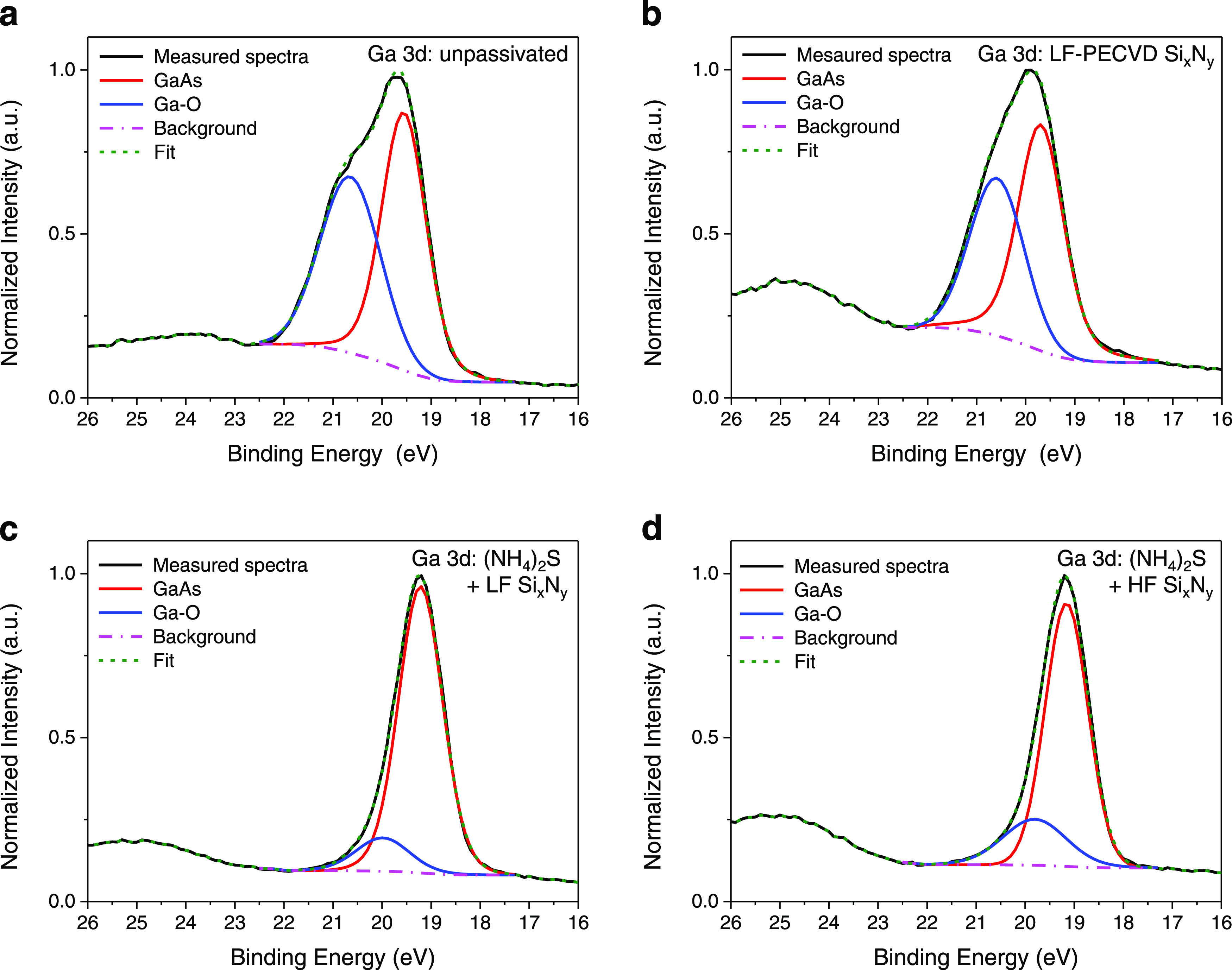
XPS spectra of Ga 3d. (a) Unpassivated sample. (b) Sample coated using LF-PECVD Si*_x_*N*_y_*. (c) Sample using ammonium sulfide treatment followed by LF-PECVD Si*_x_*N*_y_* coating. (d) Sample using ammonium sulfide treatment followed by HF-PECVD Si*_x_*N*_y_* coating.

Figure 4 shows the Ga 3d XPS spectral comparison for an untreated sample (Figure 4a) and for samples using various Si*_x_*N*_y_*-based surface treatments (Figure 4b–d). First, we analyze the passivation using LF-PECVD Si*_x_*N*_y_* without any sulfurization pretreatment. In the unpassivated case (Figure 4a), we observe a high-energy shoulder that is less pronounced for LF-PECVD Si*_x_*N*_y_* (Figure S4b). This indicates suppression of the Ga native oxide (Ga-O) peak (binding energy ∼20 eV, blue trace), showing that LF-PECVD without pretreatment already provides an impact on the removal of gallium oxides. Noteworthy, this effect is noticeable even in the case of a thin deposited layer (∼4 nm). We note that this thin layer was a requirement in our experiments to be able to perform the XPS analysis.

Next, we compare LF-PECVD Si*_x_*N*_y_* treatment with HF-PECVD Si*_x_*N*_y_* (Figure 4c,d). Clearly, in both the cases, the GaAs peak (binding energy ∼19.2 eV) is the prominent peak whereas Ga native oxides (Ga-O) are insignificant. This shows the success of combining ammonium sulfide and Si*_x_*N*_y_* coatings for the removal of native oxides. Analyzing both the cases in more detail, we observe a broader and larger Ga-O peak for the HF-PECVD Si*_x_*N*_y_*-coated sample (panel (d)), as compared to the LF-PECVD Si*_x_*N*_y_*-coated sample (panel (c)). This indicates a better performance of LF-PECVD Si*_x_*N*_y_*. These results are confirmed in Table S1 (Supporting Information S7) that summarizes the ratio of the atomic percentage of Ga-O to GaAs. A remarkable low atom % ratio (∼0.1) is achieved for the LF-PECVD Si*_x_*N*_y_-*coated sample (as compared with an atom % ratio of ∼0.3 for HF-PECVD Si*_x_*N*_y_*), which indicates the least presence of Ga-O, in line with the improvements measured in PL. A similar analysis of arsenic oxide (As-O) peaks was performed for the As 3d XPS spectra (see Supporting Information S7). As discussed in Figure S6 and Table S2, a complete suppression of native As-O oxides is achieved using ammonium sulfide combined either with LF-PECVD or with HF-PECVD Si_x_N_y_, which is in line with the trend observed in our PL measurements showing the best PL improvements for these treatments.

Following the PL results and XPS analysis, we attribute the success of our best treatments as the combined effect of three crucial factors: first, sulfide ammonium with immediate coating enables to remove native oxides and protects the surface from further reoxidation; second, additional native oxide removal using PECVD coating of Si*_x_*N*_y_* is achieved by the high level of hydrogen injection provided by plasma dissociation of SiH_4_ and NH_3_, making H^+^ the most concentrated ion in the plasma (such a mechanism has also previously been argued to be responsible for improved passivation^39^), and third, the low-frequency PECVD increases ion bombardment as H^+^ ions that are able to follow the excitation RF signal and reach the substrate surface after plasma ignition, which is able to further remove the presence of surface states. We note that the kinetic energy of ions, particularly hydrogen, gets significantly higher under the ion transit low frequency (typically below 2 MHz), resulting in ions that are able to follow the RF excitation, which then immediately reach the surface after plasma ignition. For example, recent work on metal–insulator–semiconductor capacitors fabricated by depositing Si*_x_*N*_y_* on *n*-doped GaAs at a frequency of 90 kHz^38^ reports a low density of surface states (in this case ∼10^11^ cm^–2^ eV^–1^) due to the intense ionic bombardment related to the low-frequency RF excitation.

Noteworthy, the fact that the deposition of other dielectrics (here SiO*_x_*) known to contain significant hydrogen was not found to result in similar passivation improvements strongly suggests that more than simply hydrogenation occurs. Therefore, it is possible that the Si*_x_*N*_y_* film additionally participates directly in the formation of interfacial bonding at the GaAs surface, either supplementing or substituting the existing S-terminated bonds in such a way as to reduce the density of defect sites. However, we note the exact mechanisms of the passivation effect under RF excitation in the properties of the Si*_x_*N*_y_*/GaAs interface could be further thoroughly investigated. For example, the energy of H^+^ ions that reach the sample can increase the surface temperature and stimulate surface diffusion,^42^ which can promote chemical reconstruction, leading to thermodynamically stable films. In this case, further methods such as transmission electron microscopy can be used to analyze the impact of the LF-PECVD method on the structural and morphological changes occurring on the surface of passivated GaAs.

### Time-Resolved PL Spectroscopy

**Figure 5:**
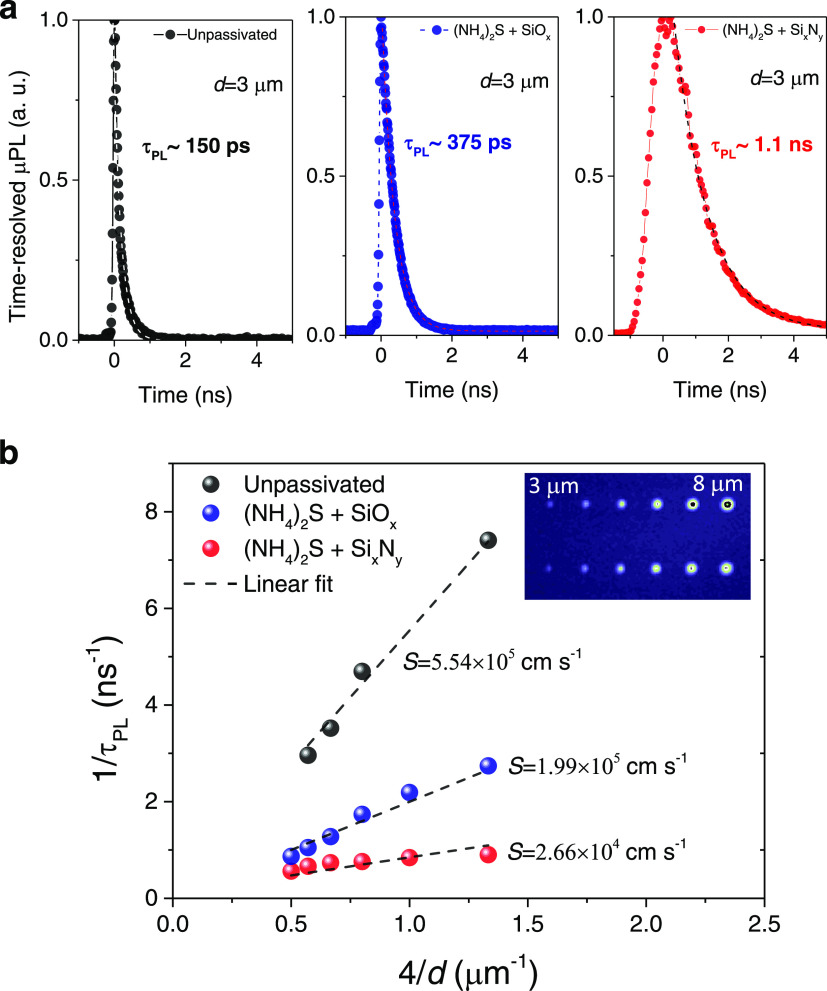
Experimental TRPL decay curves of micropillars measured at room temperature. (a) TRPL decay curves measured for a representative 3 μm wide micropillar using the treatments: (left): unpassivated (gray dots); (center): passivated pillars with SiO*_x_* coating (treatment #2, purple dots); (right): passivated pillars with Si*_x_*N*_y_* coating by low-frequency PECVD (treatment #5, red dots). The TRPL decay time is quantified using the 1/e method (dashed lines). (b) Inverse carrier lifetime, 1/τ_PL_, estimated from the TRPL measurements versus the inverse pillar width, 4/*d*, before passivation (gray dots), using SiO*_x_* coating (blue dots), and using Si*_x_*N*_y_* coating (red circles). Also shown is the corresponding linear fit (dashed black curves) of the experimental data for estimation of the corresponding surface recombination velocity, *S*. Inset shows a microscopic intensity image of the measured micropillar array for the SiO*_x_* passivated sample with pillar widths ranging from 3 and 8 μm.

To investigate the carrier dynamics in GaAs/AlGaAs pillar structures, we performed time-resolved photoluminescence spectroscopy (TRPL) measurements using a time-correlated single-photon counting (TCSPC) setup (Supporting Information S5). Due to the expected extremely short lifetimes (≪100 ps), specifically for the smaller size unpassivated pillars,^16^ and given the limited time resolution of our fastest detectors (∼50 ps)—Supporting Information S4, Figure S1—we start our analysis by first comparing the micropillar devices (≥3 μm) where the measured lifetimes are well above this limit. In Figure 5a, the measured decay curves are shown for micropillars (*d* = 3 μm) from an unpassivated (black dots trace) and two passivated samples (treatments #2 and #5, blue and red dot traces, respectively). The TRPL decay curves are fitted using a single exponential decay function to obtain the values of the carrier recombination lifetime. The results show an extremely short lifetime <150 ps for the unpassivated pillar and a lifetime ∼375 ps for the case of the passivated pillar using sulfurization followed by SiO*_x_* coating. These results are in agreement with previous measurements using identical GaAs pillar structures and similar passivation methods.^43^ Noticeably, for the case of the best passivated sample using low-frequency PECVD deposition of Si*_x_*N*_y_* (Figure 5a, right, red dot trace), the lifetime increases substantially to a value >1 ns.

To quantify the surface recombination velocity, *S*, of the measured pillars, we assume that under the low excitation conditions employed in the experiments, the surface-related nonradiative recombination rate scales as 4*S*/*d*,^37^ so that *S* can be estimated directly from the size-dependent carrier lifetimes in the low injection regime
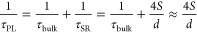
1where τ_bulk_ is the carrier lifetime in the bulk material. For nanoscale devices, generally τ_bulk_ ≫ τ_SR_, the bulk contribution can be neglected, and τ_PL_^–1^≈ 4*S*/*d*. This allows us to directly convert the measured lifetime to the surface recombination lifetime. Figure 5b shows the inverse carrier lifetime estimated from the TRPL measurements versus the inverse pillar width (4/*d*), before and after the passivation treatments presented in Figure 5a. The corresponding linear fit (dashed black curves) of the experimental data (Figure 5b) allows us to estimate a surface recombination of 5.54 × 10^5^ cm s^–1^ for unpassivated samples, and of 2.66 × 10^4^ cm s^–1^, that is, a 20-fold improvement for the LF-PECVD Si*_x_*N*_y_*-treated samples, which is in line with the trend observed in the PL measurements.

We note in our results that the decay curves for unpassivated or poorly passivated samples are exponential, and therefore, the surface-dominated recombination still remains valid. However, particularly for the best passivated samples (e.g., Figure 5a (right)), the PL can also exhibit a nonexponential decay, even when very low pumping conditions are employed. This has been reported not only for GaAs semiconductors (e.g., nanowires^27^) but also for InGaAs^37^ and InGaAsP^36^ nanostructures. Typically, this nonexponential behavior is attributed to the radiative recombination of mobile charges, which is a bimolecular process (∝*BN*^2^, where *B* is the bimolecular recombination coefficient and *N* is the photoexcited carrier density). This leads to the assumption that the initial decay curve is dominated by radiative recombination and then decays to the surface-dominated decay rate toward longer photon arrival times (specifically for highly passivated samples). Effectively, the decay curves can be modeled taking into account both surface and bimolecular recombination.^36^ However, there is still a debate on the exact phenomena that can contribute to the nonexponential behavior, which can be strongly dependent, among other factors, on the semiconductor material under study.^44^ For example, other recombination mechanisms such as trap-assisted nonradiative charge recombination, formed, for example, by defects, impurities, and dangling bonds, or inhomogeneous distribution of trap energy, can contribute to this behavior.^30^ The main goal of this paper is not to study all mechanisms of charge recombination, and therefore, for simplicity of analysis and to better compare our results with the literature, here, the data presented is quantified using a single exponential fit since the weight of the second component is rather small and therefore has a negligible contribution to the calculated lifetimes.

**Figure 6:**
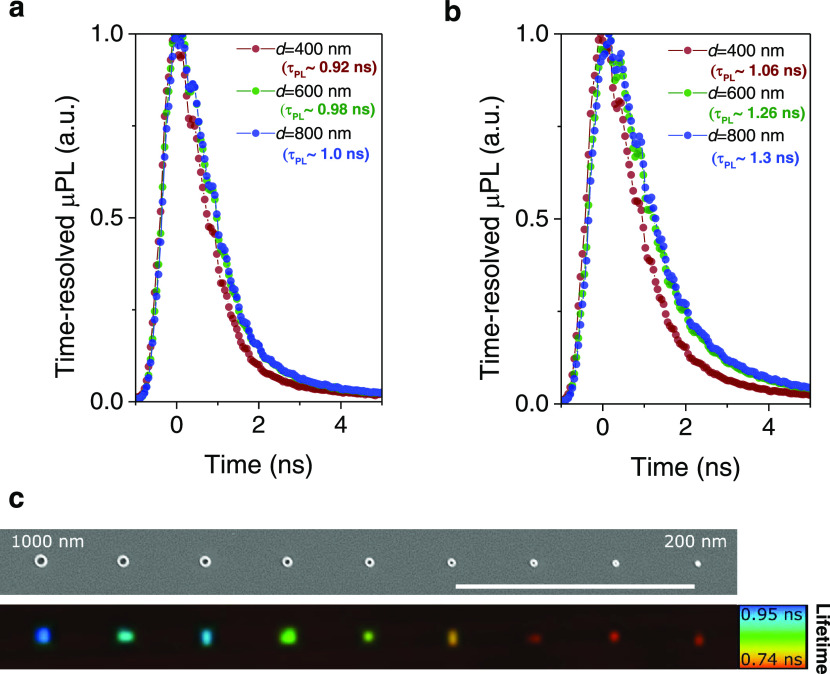
Experimental TRPL decay curves of nanopillars measured at room temperature for the best passivated samples using Si*_x_*N*_y_* coating by low-frequency PECVD (treatment #5). (a) TRPL decay curves under low pumping conditions (pump fluence ∼ 1.5 μJ cm^–2^). (b) TRPL decay curves under moderate pumping conditions (pump fluence ∼ 40 μJ cm^–2^) showing nanosecond lifetimes for nanopillars. The TRPL decay time in all plots is quantified using the 1/e method. (c) Fluorescence Lifetime Imaging (FLIM) as a function of pillar diameter for pillars ranging from 1 μm to 200 nm and under the same pumping conditions as presented in panel (b). On top of the FLIM map, a SEM image of the nanopillars is shown (scale bar is 30 μm).

Next, we analyze the TRPL decay curves of sub-micrometer pillars for the best sample in treatment #5 (Si*_x_*N*_y_* coating by LF-PECVD) that shows a remarkable 29-fold increase of PL (Figure 3a). Figure 6a shows the decay curves for a few representative pillars with 400, 600, and 800 nm pillar widths. The PL lifetimes increase from 0.92 to 0.98, and 1 ns, respectively. We note in all other measurements of poorly passivated samples (not shown), the measured lifetimes were well below the instrument response function of our setup (Supporting Information S4, Figure S1), and therefore, their lifetimes are expected to be extremely short (≪100 ps). Applying τ_PL_^–1^ ≈ 4*S*/*d*, as described previously, the calculated surface velocity recombination for the best passvated nanopillars ranges from 1.1 × 10^4^ to 2 × 10^4^ cm s^–1^. These results indicate a record-low surface velocity for dry-etched GaAs-based nanopillars, which is comparable to the best core–shell passivated GaAs nanowires^27^ of comparable width dimensions (*S* in the range of 1.7 × 10^3^ to 1.1 × 10^4^ cm s^–1^). However, these methods require challenging and expensive epitaxial growth methods. Our results show substantial improvements as compared to other methods such as doping of GaAs nanowires^16^ for enhanced radiative efficiency (*S* ∼ 2.18 × 10^6^ cm s^–1^).

We note that by increasing the pump conditions from low pumping with a pump fluence of ∼1.5 μJ cm^–2^ (Figure 6a) to a midpump fluence of ∼40 μJ cm^–2^ (Figure 6b), nanopillars can exhibit lifetimes longer than 1 ns and therefore lifetimes comparable with micropillar-sized structures. Figure 6c presents an overview of all nanopillars by showing the corresponding PL lifetime images of the nanopillars ranging from 200 to 1000 nm under the same pumping conditions as presented in Figure 6b. A clear contrast to the background of the sample is achieved, indicating that effectively the measured lifetimes are a result of the successful passivation of the pillars’ surface. The lifetimes range between 0.74 ns for the smallest nanopillars (200 nm) up to ∼0.95 ns for the 1 μm pillar size. The lifetimes exhibit slightly shorter values than the ones presented in the single histogram results (Figure 6b), which is related to lifetime binning used in the image analysis (as exemplified in Supporting Information S4, Figure S2).

### Internal Quantum Efficiency

**Figure 7:**
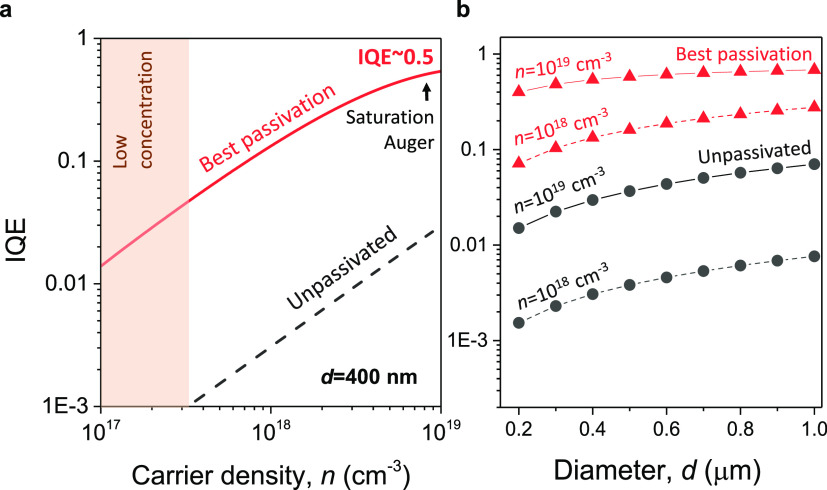
Theoretical internal quantum efficiency of nanopillars. (a) Estimated IQE for unpassivated and passivated pillars using the case of a representative pillar width of *d* = 400 nm. (b) IQE versus pillar diameter taken at carrier densities of 10^18^ and 10^19^ cm^–3^ for both unpassivated and passivated pillars. All plots assumed the Auger recombination effect using typical values for GaAs material, *C* = 3.5 × 10^–30^ cm^6^ s^–1^.

To further characterize the effect of the large reduction of the surface recombination velocity on the internal quantum efficiency (IQE) of pillars, we have calculated the IQE, which is the ratio of the radiative emission rate (τ_*r*_^–1^= *Bn*^2^) to the sum of nonradiative and radiative emission rates (τ_nr_^–1^ + τ_*r*_^–1^), for the case of a 400 nm pillar width for both the best passivated (*S* = 1.1 × 10^4^ cm s^–1^) and unpassivated (*S* = 5.54 × 10^6^ cm s^–1^) scenarios. In this analysis, Auger recombination, *C*,^45^ was also considered so that the nonradiative term reads τ_nr_^–1^ = (4*S*/*d*)*n* + *Cn*^3^. As displayed in Figure 7(a), in the low concentration regime (carrier density of 3 × 10^17^ cm^–3^), IQE values of ∼0.04 are obtained for the passivated case, while this value drops substantially to an IQE of 9.2 × 10^–4^ in the case of the unpassivated sample. Remarkably, for a larger carrier density concentration (10^19^ cm^–3^), where nanoLEDs are expected to operate,^46^ a high value of IQE = 0.54 is calculated for the 400-nm-sized pillar, limited only by Auger recombination. This is a 20-fold improvement as compared to the unpassivated nanopillar (IQE ∼ 0.028). This analysis illustrates the strong role of nonradiative effects on the low efficiency reported in III–V nanolight sources.^3,10,11^ As shown in Figure 7b, the IQE for the Si*_x_*N*_y_* passivation case remains high (≥0.1) for all analyzed pillar sizes (0.2 μm to 1 μm) and for the carrier density values considered (10^18^ and 10^19^ cm^–3^). We note these results indicate a best case scenario and do not take into account other factors that can play a role in the efficiency of nanopillar devices, such as carrier injection efficiency. The results reported here, combined with the enhancement of the light extraction efficiency for identical sub-micrometer GaAs/AlGaAs pillars reported elsewhere,^43^ could lead to substantial improvements of the external quantum efficiency of nanostructures such as nanopillars or nanorods, for example, when integrated in photonic crystal cavities, optical resonators, or coupled to waveguides, etc. Therefore, our findings are of key importance for the miniaturization of GaAs optical components and devices.

## Conclusions

We have successfully passivated the surface of GaAs/AlGaAs nanopillars using a combination of ammonium sulfide chemical treatment followed by encapsulation with silicon nitride, a widely used dielectric, by low-frequency plasma deposition. We demonstrate up to a 29-fold increase of the photoluminescence integrated intensity at room temperature for the best passivated nanopillar samples as compared to unpassivated nanopillars. This leads to estimations of a low surface velocity of ∼1.1 × 10^4^ cm s^–1^ for dry-etched GaAs-based nanopillars. The wide range of tests and analysis performed, including XPS analysis to investigate the amount of surface defects, confirm that the best passivation treatment is a combination of three crucial factors: first, sulfurization of GaAs surfaces with immediate coating enables to remove native oxides without further reoxidation. Importantly, sulfurization prepares the initial surface for the coating material; second, additional native oxide removal using PECVD coating of Si*_x_*N*_y_* is achieved by the high level of hydrogen injection; and third, the low-frequency (380 kHz) plasma enables intense ionic bombardment of H^+^ ionic species as a result of the RF excitation, playing an active role in the passivation of nanopillars by further removing the presence of surface states. We note that according to previous studies,^38^ in principle, the low-frequency effect should be observed for a wide range of frequencies of the plasma as long as the selected frequency is under the ion transit low frequency (typically below 2 MHz). Since our PECVD system uses two fixed RF power generators at 380 kHz and 13.56 MHz, it was impractical to implement further studies with varying frequencies. Taking advantage of this passivation method, the low-frequency plasma shows a unique potential to be used in industrial environments as a highly reproducible and cost-effective passivation method needed for the exponentially growing miniaturized GaAs devices and applications, namely, electrically pumped nanopillar LEDs. Since GaAs-based devices typically require post rapid annealing temperature treatments for the annealing of electrical contacts, we identify that further studies on the stability of the passivation would be relevant. In fact, several studies show that passivation treatments benefit substantially from post-annealing,^36^ which could improve the results achieved here. Importantly, the passivation method based on low-frequency PECVD can potentially be extended to other III–V materials covering additional wavelengths and be exploited for a wide range of high-performance room-temperature nano-optoelectronic active devices such as nanoLEDs, nanolasers, nanophotodetectors needed for energy-efficient emerging photonic integrated circuit technologies, with applications in neuromorphic or quantum photonic computation, bioimaging, information and communication technologies and internet of things, or improved performance of nanostructured solar cells.

## Supporting Information Available

Fabrication of GaAs/AlGaAs micro- and nanopillars; surface passivation treatments; micro-photoluminescence (μPL) characterization; time-resolved micro-PL; internal quantum efficiency; PL of bulk GaAs material; energy-dispersive X-ray spectroscopy; and X-ray photoelectron spectroscopy (PDF)
