# Supplementary material for: Surface Passivation of III–V GaAs Nanopillars by Low-Frequency Plasma Deposition of Silicon Nitride for Active Nanophotonic Devices
Source: ACS Appl Electron Mater. 2022 Jul 1;4(7):3399–410. doi: 10.1021/acsaelm.2c00195 (PMC9778088; doi:10.1021/acsaelm.2c00195)
Supplement: Supplementary file 1 — el2c00195_si_001.pdf [file el2c00195_si_001.pdf]

Supporting Information

Surface Passivation of III-V GaAs Nanopillars by Low Frequency Plasma Deposition of Silicon Nitride for Active Nanophotonic Devices

Bejoys Jacob[1]‡, Filipe Camarneiro[1]‡, Jérôme Borme[2], Oleksandr Bondarchuk[3], Jana B. Nieder[1]**, and Bruno Romeira[1]*

[1]INL – International Iberian Nanotechnology Laboratory, Ultrafast Bio- and Nanophotonics group, Av. Mestre José Veiga s/n, 4715-330 Braga, Portugal

[2]INL – International Iberian Nanotechnology Laboratory, 2D Materials and Devices group, Av. Mestre José Veiga s/n, 4715-330 Braga, Portugal

[3]INL – International Iberian Nanotechnology Laboratory, Advanced Electron Microscopy, Imaging and Spectroscopy Facility, Av. Mestre José Veiga s/n, 4715-330 Braga, Portugal

Corresponding Authors

*bruno.romeira@inl.int, **jana.nieder@inl.int

‡ These authors contributed equally

S1. Fabrication of the GaAs/AlGaAs micro- and nanopillars

The fabrication used samples cleaned first using acetone and isopropanol (IPA) to remove the photoresist layer followed by 13 minutes oxygen plasma at 230 W to remove any organic material and followed by a deoxidation treatment of 2 minutes in diluted NH_4OH:H_2O (1:10). The fabrication of the micro- and nanopillars involved an electron beam lithography (EBL) step using a 5200 ES 100 kV tool from Vistec, where the pillars were patterned using a negative e-beam resist (ARN7520.18) of 500 nm thickness with a 200 nm SiO_x hard mask deposited by plasma enhanced chemical vapor deposition [PECVD (model CVD MPX, a machine from SPTS)] at 300°C. Before the resist deposition a treatment of hexamethyldisilazane (HMDS), a primer deposited in an oven at 150 ºC was used to favor uniformity and adhesion in the resist deposition. After exposure of the resist using EBL, baking on a hot plate at 85 °C for 60 s is performed, followed by a 120 s of development using developer AR 300.47 (TMAH) diluted 4:1 in water. The sample is cleaned with water and the developer solution refreshed every 30 s. The sample is finally spin dried. The next step involved transferring the pattern from the resist to the hard mask using a module for reactive ion etching (APS, a machine from SPTS) (etch rate of ~594 nm/min). Once the pattern was transferred, the remaining resist was removed by 13 minutes oxygen plasma at 230 W followed by a deoxidation step of 2 minutes in diluted NH_4OH:H_2O (1:10). The sample was then rinsed in ultra-pure water (UPW) and dried with an N_2 pistol. The following step was to etch the pillars by dry etching with inductively coupled plasma (ICP, a machine from SPTS) using a mixture of Ar and BCl_3 chemistry at 40°C to deeply etch the pillars until ~0.54 μm depth, followed by a cleaning step were the samples were rinsed in UPW and dried with an N_2 pistol. After, the remaining SiO_x hard mask was etched with hydrofluoric (HF) acid in a vapor etcher tool (Primaxx uEtch, a machine from SPTS) using anhydrous HF vapor at 13% during 600s, followed by one cycle of 13 minutes

oxygen plasma treatment at 230 W to clean the surface of the pillars. The last step was a deoxidation step using a solution of $NH_4OH:H_2O$ (1:10) for 2 min. After, the samples were rinsed in UPW and dried with an N_2 pistol.

S2. Surface passivation treatments

After fabrication of the nanopillars, the surface passivation procedures consisted in the following six main treatments.

Surface treatment #1: $(NH_4)_2S$ chemical treatment. The samples, previously treated diluted in $NH_4OH:H_2O$ (1:10) were after immediately treated with a sulfur treatment consisting of ammonium sulfide as a passivation agent, where the samples were submerged in a diluted solution of $H_2O:(NH_4)_2S$ (1:10) for 5 minutes at 65 °C under dark conditions, prepared using an ammonium sulfide solution, 20% in H_2O. The samples were then dried with an N_2 pistol, without rinsing in between.

Surface treatment #2: $(NH_4)_2S$ + SiO_x coating by HF-PECVD. The samples were submerged in ammonium sulfide solution as described in treatment 1. For the dielectric coating step, immediately after the sulfur treatment (less than 5 minutes) a thin capping layer of SiO_x of 100 nm was deposited by high-frequency (13.56 MHz) PECVD (deposition time of 126s), using 1420:10:392 sccm $N_2O:SiH_4:N_2$ as precursor gases at a pressure of 900 mTorr and power of 30 W. The deposition of non-stoichiometric SiO_x was done at 300°C covering the walls and the top of the pillars.

Surface treatment #3: $(NH_4)_2S$ + SiO_x coating by LF-PECVD. In this treatment the samples were submerged in ammonium sulfide solution as described in treatment 1, immediately followed by a thin capping layer of SiO_x deposited by low-frequency (380 kHz) PECVD. The precursor gases used here consists of using 1420:12:392 sccm $N_2O:SiH_4:N_2$ as precursor gases at a pressure of 900 mTorr and power of 60 W. The deposition of non-stoichiometric SiO_x was done at 300°C covering the walls and the top of the pillars.

Surface treatment #4: $(NH_4)_2S$ + Si_xN_y coating by HF-PECVD. Immediately after submerged in ammonium sulfide the samples were coated by a layer of Si_xN_y deposited by high-frequency (15.56 MHz) PECVD at 300°C. The precursor gases used here consisted of 40:55:1960 sccm SiH_4:NH_3:N_2 at a pressure of 900 mTorr and power of 30 W.

Surface treatment #5: $(NH_4)_2S$ + Si_xN_y coating by LF-PECVD. The samples were submerged immediately in ammonium sulfide and then coated by a thin layer of Si_xN_y of 81 nm deposited by low-frequency (380 kHz) PECVD (deposition time of 120 s). The precursor gases used here consists of 40:20:1960 sccm SiH_4:NH_3:N_2 at a pressure of 550 mTorr and power of 60 W. The deposition of non-stoichiometric Si_xN_y was done at 300°C.

Surface treatment #6: Si_xN_y coating only by LF-PECVD. In this treatment, the samples were not submerged in ammonium sulfide solution previously to dielectric coating. Instead, the samples previously treated diluted in NH_4OH:H_2O (1:10) were after immediately coated with a thin capping layer of Si_xN_y deposited by low-frequency (380 kHz) PECVD, using the same dielectric deposition conditions as described in surface treatment #4.

S3. Micro-photoluminescence (μPL) characterization

The emission spectral intensity of micro- and nanopillars from the GaAs/AlGaAs layer stack semiconductor material was collected using a micro-photoluminescence microscope integrated with a spectrometer covering the visible and near-infrared region of the spectrum. It consists of a confocal Raman system in PL mode (WITec Alpha300M+, a tool from Witec Ulm) equipped with a 100× air high numerical aperture objective (NA=0.9). We have used a continuous-wave laser at 532 nm excitation wavelength (power level <100 μW) to pump the micro-and nanopillars. The collected light was filtered with a 532 nm bandpass filter and focused to a multimode fiber. The fiber-coupled light is then sent to a UHTS300 spectrometer (with 600 lines/mm grating) and then coupled to an Andor Peltier cooled CCD detector.

S4. Time-resolved micro-PL

In the time-resolved PL measurements, excitation from a pulsed laser diode (~561 nm)(BDL-561-SMY, Becker & Hickl) was used with a pulse width ~80 ps, a repetition rate of 50 MHz and a pump fluence of ~1.5 μJ/cm^2. The laser pulses are guided into a custom-built microscope based on an inverted microscope design by steering silver galvo-scanner mirrors, and expanded by a set of scan and tube lenses (SL50-CLS2 and TTL200MP, Thorlabs). The sample scanning is done via the aforementioned galvo-scanning mirrors changing the laser angle at the objective back aperture, while the sample positioning and fine focus are done via a manual XY micrometer stage and a nanometer Z-piezo stage (Nano-Z100-N, MadCityLabs). The light emitted from the pillars was collected by an oil immersion 100× high-numerical aperture objective (PFO 100x 1.3NA, Nikon). The collected light was guided to a single-photon counting avalanche photodetector (APD) (QD800c-fQ, Roithner Lasertechnik) to measure the temporal decay. For unpassivated samples showing extremely short lifetimes, see Fig. 1(a), a fastest APD (PD50CTD, MPD) was used, see respective instrument response functions (IRFs) in Fig. S1. Prior to detection, a long pass spectral filter (~561 nm) was used to select the signal of interest and filter out unwanted background signals. The APD was connected to a correlation card (TCSPC 150N, Becker & Hickl) controller. This controller correlates the photon arrival times at the APD (start signal) with the electric laser pulse arrival times (stop signal) in order to measure the luminescence decay. A histogram of these arrival times is then constructed corresponding to the time-dependent output intensity of the optically pumped pillars. The produced lifetime data was analyzed via the SPCImage software (Becker & Hickl) and via Origin using single fit algorithms. Even though some decay curves exhibit double exponential behavior, the weight of second component is rather small and therefore has a negligible contribution to the calculated lifetimes. Figure 5(b) in the paper shows a SEM image and a FLIM

image of the nanopillars ranging from 200 to 1000 nm. The lifetime analysis shows a clear size dependency of the nanopillars lifetime. The lifetime values exhibit relatively shorter values than the ones presented in the single histogram results due to lifetime binning used in the image analysis. This binning takes into account the nearest neighboring pixels to calculate the lifetime of each pixel, increasing the number of counts per decay curve and improving the signal to noise ratio. The shorter lifetime values can be explained by the heterogeneous lifetime distribution inside the pillars, as exemplified for the case of micropillars (see Fig. S2), where the center of the pillars exhibit longer lifetimes than the border.

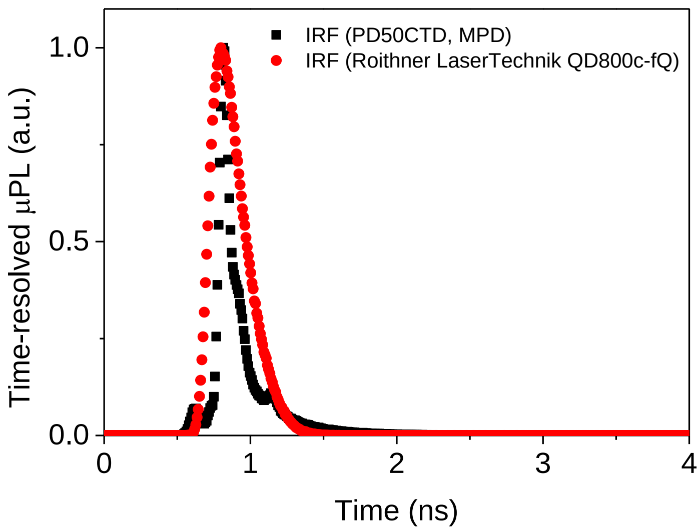

Figure S1: Instrument response functions of both QD800c-fQ (Roithner Lasertechnik), and PD50CTD (MPD) detectors.

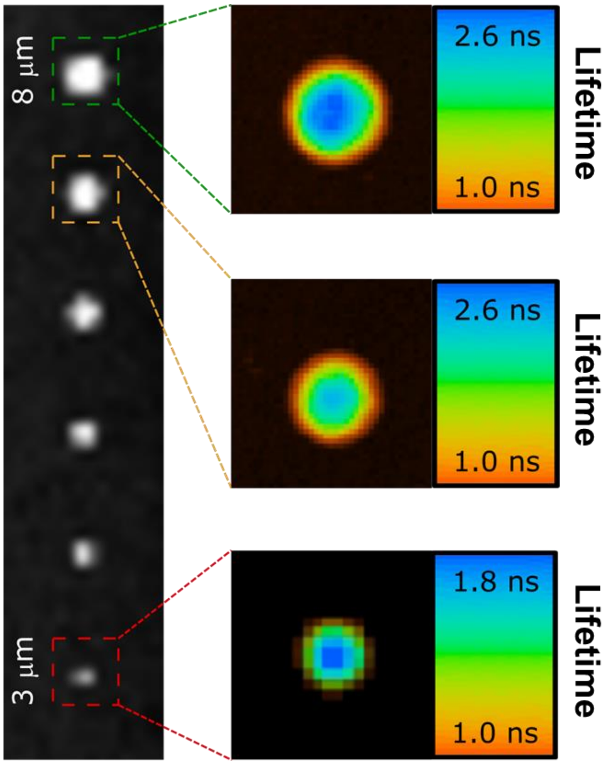

Figure S2: Intensity (left) and FLIM images (right) of micropillars ranging from 8 to 2 µm, taken with a 561 nm ps-laser. FLIM scans show a heterogeneous distribution of the lifetime within the micropillars. The center of the pillars exhibit longer lifetimes in comparison with the borders.

S5. PL emission of bulk GaAs surrounding the etched pillars

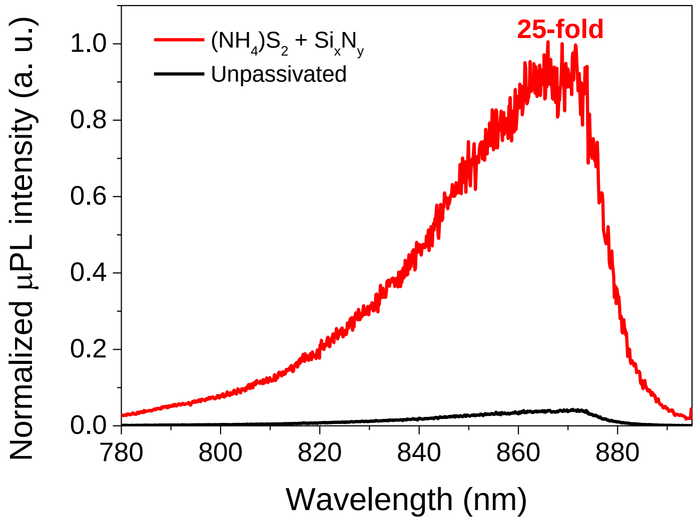

Figure S3: Steady state photoluminescence spectra measured at room-temperature displaying a typical µ-PL spectra from the etched bulk GaAs material surrounding the etched pillars. The spectra show the band-edge emission at ~872 nm, typical of a bulk GaAs material with a clear effect of the best passivation treatment displaying a 25-fold enhancement of the integrated PL emission. The results compare well with the typical improvements observed in nanopillars.

S6. Energy-dispersive X-ray spectroscopy (EDS)

Experiment

For initial surface characterization studies using energy-dispersive X-ray spectroscopy (EDS), we used a scanning electron microscope (SEM), (FEI NovaNanoSEM 650), equipped with an EDS system (Oxford x-act). The system was operated using a voltage of 5 kV. The identification of spectral lines was performed using INCA software.

Results and discussion

Figure S4 shows the EDS analysis of measured pillars (pillar width ranging from 200 nm – 1 μm) for the best treatments shown in PL measurements employing Si_xN_y coating layers. The results in both panels (a) and (b) do not show traces of oxygen indicating good passivation treatments. We note however the EDS analysis in our SEM system is challenging to quantify native oxides below one atomic percentage (at %), in particular for light atoms. Further, for reproducible comparison of results a good reference sample in the same percentage range as the expected change of composition is typically required. As a result, to quantify the removal of gallium and arsenic oxides we focused our attention on samples measured by X-ray photoelectron spectroscopy (XPS), see next section S7. We note in Fig. S4(a) we see traces of adventitious carbon. We attribute this to the fact that measurements were realized in samples after more than 12 months of the passivation being performed.

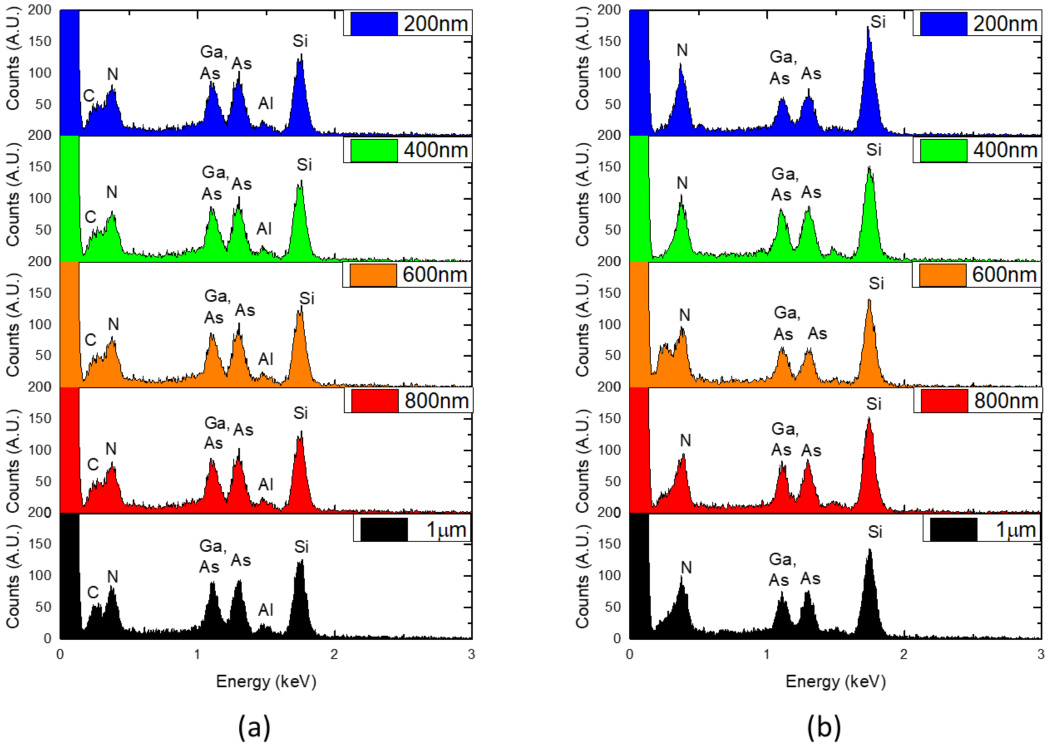

Figure S4: EDS spectra for nanopillars varying with size ranging from 200 nm-1 μm. (a) Sample coated with LF-PECVD Si_xN_y without sulfurization. (b) Sample pre-treated with ammonium sulfide and coated with LF-PECVD Si_xN_y. (In all measurements a voltage of 5 kV was used).

S7. X-ray photoelectron spectroscopy (XPS)

Experiment

For further explaining the potential of the LF-PECVD Si_xN_y to passivate GaAs, additional measurements using X-ray photoelectron spectra (XPS) were performed. Considering the typical analyzed large area covered by our XPS ssytem (650 μm × 650 μm), for the measurements additional samples of ~500 nm etched GaAs (without patterned pillars) were prepared, following the same etching procedure conditions (see S1) as performed for the nanopillars. The remaining passivation treatments followed identical conditions as discussed earlier (section S2) and here we focused only on samples using the Si_xN_y-based best treatments: (a) unpassivated sample, (b) sample with LF-PECVD Si_xN_y coating only (treatment #6), (c) sample with ammonium sulfide passivation and coating of Si_xN_y by LF-PECVD (treatment #5), (d) sample with ammonium sulfide passivation followed by coating using HF-PECVD Si_xN_y (treatment #4). The key difference of the treatments employed is that since XPS spectra can effectively measure only thicknesses within 10 nm from the surface, for these samples a thickness of only ~4 nm of Si_xN_y (instead of ~80 nm) was deposited coating the etched GaAs surface. The XPS spectra was collected using an ESCALAB 250Xi system (Thermo Scientific) in UHV (< 10^{-9} Torr). The monochromatic Al-Kα source (1486.6 eV) was used to analyze an area of 650 μm × 650 μm in the prepared samples. For fitting the peaks of measured spectra we used Avantage software with Voigt functions (convolution of Lorentzian and Gaussian functions). As discussed next the peak fittings closely match the standard chemical states previously recorded in literature.[1,2] The calibration of the peaks was done by adventitious carbon peak (284.8 eV).

Results and discussion

Unlike other semiconductors, as for example silicon, the native oxides formed at the surface of

GaAs are not stable. This contributes to non-radiative recombination sites at the GaAs surface limiting the PL of the GaAs material.[3] Through passivation methods, the removal of these oxide layers and respective replacement with stable coating material is achieved. Thus the quality of the passivation treatment can be quantified by the minimum amount of surface defects formed by the native oxides of GaAs (here Ga-O (Ga_2O_3) and As-O (As^{3+} and As^{5+})). Here we focus our analysis on the removal of these native oxides using the best treatments shown in PL measurements employing Si_xN_y coating layers.

Figure S5 (identical figure as Fig. 4 in the main paper) shows the Ga 3d XPS spectra comparison for an untreated sample, Fig. S5(a), and for samples using various Si_xN_y-based surface treatments, Figs. S5(b)-(d). First we analyze the passivation using LF-PECVD Si_xN_y without any sulfurization pre-treatment. In the unpassivated case, spectrum of Figure S5(a), we observe a high energy shoulder which is less pronounced for the LF-PECVD Si_xN_y treatment, Figure S5(b). This indicates suppression of the Ga native oxide (Ga-O) peak (binding energy (B.E.) ~20 eV, blue trace), indicating the treatment with LF-PECVD without pre-treatment provides already an impact on the removal of gallium oxides. Noteworthy, this effect is already noticeable even in the case of a thin deposited layer (~4 nm). We note this thin layer was a requirement in our experiments to be able to perform the XPS analysis.

Next we compare the LF-PECVD Si_xN_y treatment versus the HF-PECVD Si_xN_y, Figs. S5(c) and (d), respectively. In both cases an ammonium sulfide pre-treatment was used. Clearly in both cases the GaAs peak (binding energy ~19.2 eV) is the prominent peak whereas Ga native oxides (Ga-O) are insignificant. This shows the success of combining the ammonium sulfide and Si_xN_y coatings for the removal of native oxides. When comparing in more detail both cases, we observe a broader and larger Ga-O peaks for the HF-PECVD Si_xN_y coated sample, panel (d), as compared to the LF-PECVD Si_xN_y coated sample, panel c). This indicates a better performance of the LF-PECVD Si_xN_y

cases as compared with the HF-PECVD Si_xN_y. The results are confirmed in Table S1 which summarizes the ratio of the atomic percentage (at %) of Ga-O to GaAs. A low at % ratio (~ 0.14) is achieved for LF-PECVD Si_xN_y coated sample which indicates the least presence of Ga-O for the best treatment and in line with the trend observed in PL measurements.

Table S1: Fitting parameters and atomic percentage ratio of the XPS spectra of Ga 3d.

**Treatment**	**GaAs B.E. (eV)**	**GaAs Peak FWHM (eV)**	**Ga-O B.E. (eV)**	**Ga-O Peak FWHM (eV)**	**Ratio of at % (Ga-O /GaAs)**
Unpassivated	19.55	1.07	20.65	1.45	0.88
LF-PECVD Si_xN_y only	19.69	1.1	20.59	1.36	0.78
$(NH_4)_2S$ + LF-PECVD Si_xN_y	19.21	1.06	19.99	1.28	0.14
$(NH_4)_2S$ + HF-PECVD Si_xN_y	19.16	1.03	19.81	1.53	0.27

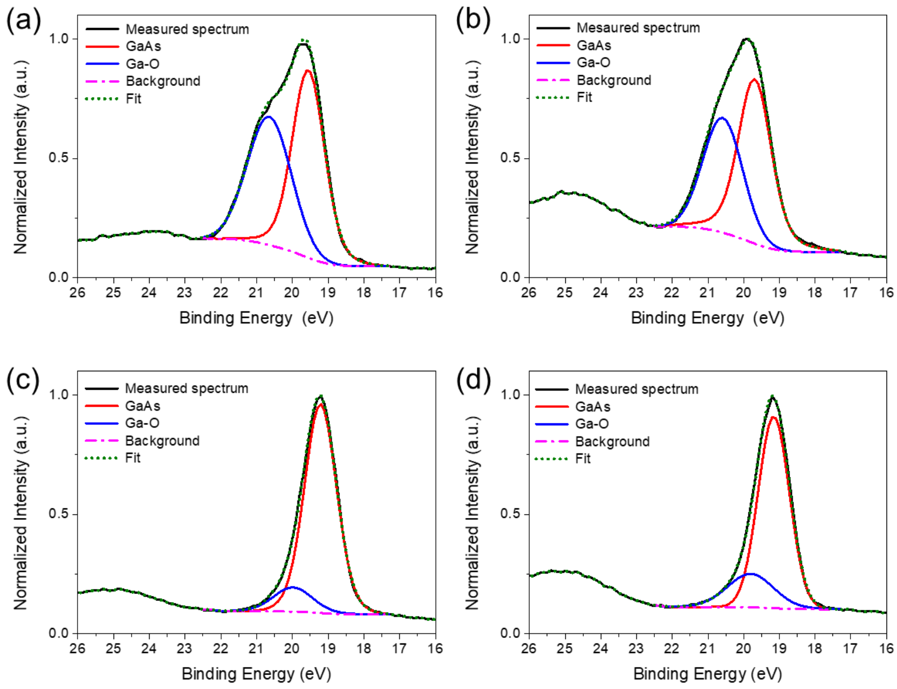

Figure S5: XPS spectra of Ga 3d for: (a) Unpassivated sample. (b) Sample coated using LF-PECVD Si_xN_y. (c) Sample using ammonium sulfide treatment followed by LF-PECVD Si_xN_y coating. (d) Sample using ammonium sulfide treatment followed by HF-PECVD Si_xN_y coating. [The results in this figure are also shown in the main paper, Fig. 4.]

In what follows we analyze the As 3d XPS spectra, Fig. S6. First we compare the unpassivated sample, panel (a), with a sample using LF- PECVD Si_xN_y treatment without sulfurization, panel (b). A shift in As-O peaks (both As^{3+} and As^{5+}) is observed for the sample with LF-PECVD Si_xN_y treatment as compared with the unpassivated sample. Further, a prominence in As^{5+} peaks (As^{5+} $3d_{5/2}$ and $3d_{3/2}$) is observed for the sample with LF-PECVD Si_xN_y treatment, panel (b), as compared to the prominent As^{3+} peaks (As^{5+} $3d_{5/2}$ and $3d_{3/2}$) for the untreated sample, panel (a). This indicates an effect of the LF-PECVD Si_xN_y treatment on the GaAs surface. Possibly the Si_xN_y film additionally participates directly in the formation of interfacial bonding at the GaAs surface.

Table S2 shows the As 3d fitting parameters and the at % ratio of As-O to GaAs. The at % ratio is 1.27 for the passivated sample, which compares with an at % ratio of 1.52 for the unpassivated sample. The lower ratio indicates oxide removal is achieved despite the very thin layer deposited. Noteworthy the samples treated with ammonium sulfide followed by the Si_xN_y deposition, Figs. S6 (c), (d), show a clear suppression of native oxides. Here, due to the successful suppression of As-O peaks in both cases the effect of the plasma frequency (LF vs HF Si_xN_y PECVD) was not possible to compare.

Table S2: Fitting parameters and atomic percentage ratio of the XPS spectra of As 3d.

**Treatment**	**GaAs $3d_{5/2}$ B.E. , FWHM (eV)**	**GaAs $3d_{3/2}$ B.E. , FWHM (eV)**	**As-O (As^{3+}) $3d_{5/2}$ B.E. , FWHM (eV)**	**As-O (As^{3+}) $3d_{3/2}$ B.E. , FWHM (eV)**	**As-O (As^{5+}) $3d_{5/2}$ B.E. , FWHM (eV)**	**As-O (As^{5+})$3d_{3/2}$ B.E. , FWHM (eV)**	**Ratio of at % (As-O/ GaAs)**
Unpassivated	41.31, 0.99	42.03, 0.99	44.45, 1.63	45.2, 1.63	45.78, 1.29	46.54, 1.29	1.52
LF PECVD Si_xN_y only	41.48, 1.06	42.19, 1.06	44.26, 1.42	44.89, 1.42	45.3, 1.49	46.1, 1.49	1.27
$(NH_4)_2S$ + LF PECVD Si_xN_y	40.91, 0.95	41.61, 0.95	N/A	N/A	N/A	N/A	N/A
$(NH_4)_2S$ + HF PECVD Si_xN_y	40.85, 0.92	41.55, 0.92	N/A	N/A	N/A	N/A	N/A

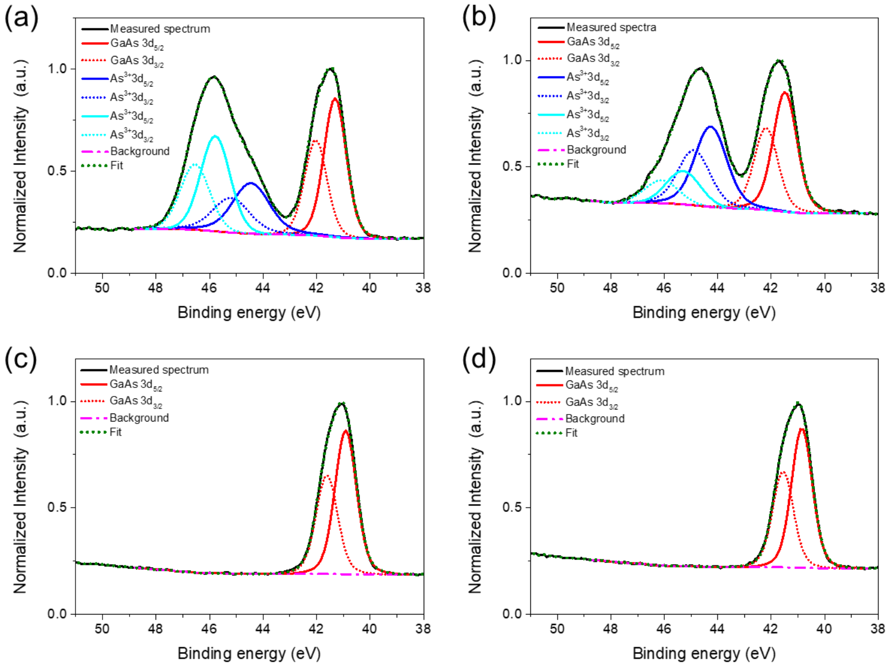

Figure S6: XPS spectra of As 3d for: (a) Unpassivated sample. (b) Sample coated using LF-PECVD Si_xN_y. (c) Sample using ammonium sulfide treatment followed by LF-PECVD Si_xN_y coating. (d) Sample using ammonium sulfide treatment followed by HF-PECVD Si_xN_y coating.

REFERENCES

(1) Ghosh, S. C.; Biesinger, M. C.; LaPierre, R. R.; Kruse, P. The Role of Proximity Caps during the Annealing of UV-Ozone Oxidized GaAs. *J. Appl. Phys.* **2007**, *101* (11), 114321.

(2) Ghosh, S. C.; Biesinger, M. C.; LaPierre, R. R.; Kruse, P. X-Ray Photoelectron Spectroscopic Study of the Formation of Catalytic Gold Nanoparticles on Ultraviolet-Ozone Oxidized GaAs(100) Substrates. *J. Appl. Phys.* **2007**, *101* (11), 114322.

(3) Richard, O.; Blais, S.; Arès, R.; Aimez, V.; Jaouad, A. Mechanisms of GaAs Surface Passivation by a One-Step Dry Process Using Low-Frequency Plasma Enhanced Chemical Deposition of Silicon Nitride. *Microelectron. Eng.* **2020**, *233*, 111398.
